# The comparison between Intrauterine Insemination and Fallopian Tube Sperm Perfusion Using FAST®System in Patients with Unexplained Infertility

**DOI:** 10.22074/ijfs.2015.4177

**Published:** 2015-02-07

**Authors:** Sepideh Peivandi, Aghdas Ebadi, Shila Modanlu

**Affiliations:** 1 Department of Gynecology and Obstetrics, IVF Unit, Imam-Khomeini Hospital, Faculty of Medicine, Mazandaran University of Medical Sciences, Sari, Iran; 2 Department of Gynecology and Obstetrics, Imam-Khomeini Hospital, Faculty of Medicine, Mazandaran University of Medical Sciences, Sari, Iran

**Keywords:** Infertility, Ovarian Stimulation, Insemination

## Abstract

**Background:**

Controlled ovarian stimulation (COH) with intrauterine insemination (IUI) is commonly offered to infertile couples with patent fallopian tubes because it is simple, non-invasive and cost-effective technique. Another non-invasive method is fallopian tube sperm perfusion (FSP). This study was performed to compare the relative efficacy between FSP using fallopian sperm transfer (FAST) system and standard IUI in patients with unexplained infertility.

**Materials and Methods:**

This prospective randomized study was conducted at the IVF Unit, Department of Gynecology and Obstetrics, Mazandaran University of Medical Sciences, Sari, Iran, from March 2011 to February 2012. A total of ninety patients with unexplained infertility underwent ovarian stimulation with clomiphene citrate and human menopausal gonadotropin (HMG). Patients were then randomly assigned into either group I (n=45) to undergo standard IUI or group II (n=45) to undergo FSP using FAST system.

**Results:**

The patients’ basic characteristics, including age, primary infertility and duration of infertility, were not significantly different between two study groups. In the group I, there were 9 pregnancies (a pregnancy rate per cycle of 20%), whereas in the group II, 8 pregnancies occurred (a pregnancy rate per cycle of 17.8%, p>0.05).

**Conclusion:**

FSP using FAST system offers no advantage over the standard IUI in order to increase pregnancy rate in patients with unexplained infertility.

## Introduction

Controlled ovarian hyperstimulation (COH) along with intrauterine insemination (IUI) is commonly used to infertile couples with patent fallopian tube. IUI is simple, non-invasive and cost-effective technique (1). During IUI, pretreated semen is concentrated in a small volume of 0.2-0.5 ml and splashed by a catheter into the uterine cavity (2, 3). Different studies have been reported a pregnancy rate per cycle of 15-20% (4-6). The pregnancy rate depends on artificial insemination technique, the type of ovarian stimulation [Clomid or injectable gonadotropins, with or without gonadotropin-releasing hormone (GnRH)], the age of patients and the cause of infertility (1).

Fallopian tube sperm perfusion (FSP), an alternative procedure, has been reported to improve pregnancy rate in comparison with IUI (7-9). The FSP was first described by Kahn et al. (7) and shown a pregnancy rate per cycle of 26.9% in patients with unexplained infertility. In FSP technique, sperm preparation is identical to that used in IUI, but the main difference is the sperm preparation volume of medium that is 4 ml in FSP, indicating higher volume in comparison with IUI (10). Therefore, higher volume of insemination in FSP technique causes sperms to pass directly through the fallopian tubes and to spread into the cul-de-sac (11). Many studies have shown the higher sperm densities in the fallopian tubes present at the time of ovulation as compared with IUI (12). In a study by Ripps et al. (13), they showed that the number of peritoneal spermatozoa recovered at laparoscopy after IUI was very less than their number after uterotubal flushes. Mamas (14) proposed 10 ml of contrast medium in hysterosalpingography is sufficient to fill the uterine cavity and to pass through fallopian tube in order to spread in peritoneal cavity, suggesting the efficiency of tuboperitoneal insemination (IUTPI) method.

There are different method to prevent semen reflux in FSP technique, such as using Allis clamp on cervix, transcervical inflated pediatric Folley catheter balloon, the double nut bivalve (DNB) speculum with modified tips to clamp the cervix, and the fallopian sperm transfer (FAST) system(1,7, 8,10,15-18). In a study by Fanchin et al. (1), they introduced the FAST system, an autoblocking device for FSP. They reported significant difference in pregnancy rate per cycle, 40% in the FSP Vs. 20% in the IUI group.

Since 1992, several randomized controlled studies published have compared the efficacy between FSP and standard IUI, but they have showed conflicting results (1, 9, 10, 17, 19). Since there was no study about FSP using FAST system in North of IRAN .We designed this prospective trial to evaluate and to compare the pregnancy rate per cycle between FSP using FAST system and IUI in patients with unexplained infertility.

## Materials and Methods

This prospective randomized study was conducted at the IVF Unit, Department of Gynecology and Obstetrics, Imam-Khomeini Hospital, Mazandaran University of Medical Sciences, Sari, North of Iran, from March 2011 to February 2012. After a basic infertility work up, patients with unexplained infertility were included in this study with the following indications: normal ovulatory cycle, normal spermiogram, normal hysterosalpingography, and normal laparoscopy finding. Patients with abnormal semen morphology and hormonal assay, abnormal hysterosalpingography, age more than 35 years, polycystic ovarian syndrome (PCOS) and endometriosis, body mass index (BMI)>28 kg/m^2^,duration of infertility>10 years, and history of treatment with assisted reproductive techniques(ART) were excluded. After obtaining informed consent, all patients underwent similar controlled ovarian stimulation protocol using clomiphene citrate and human menopausal gonadotropin (hMG) injection. The protocol consisted of clomiphene citrate (tablet 50 mg, Iran Hormone, Iran) 100 mg per day from day 3 to 7 of the menstrual cycle and a single intramuscular injection of hMG (Merional, IBSA, Switzerland) 75-150 IU daily (single dose) until the follicle diameter reach to 18 mm. Cycles were monitored from day 10 by transvaginal ultrasound (Honda 2000, japan) to measure the number and the diameter of the growing follicles and endometrial thickness. The maturation of two to three follicles was considered optimal. A total of 10,000 IU human chorionic gonadotropin (hCG, Amp 5000 IU/1 cc, Darupakhsh, Iran) was administered when at least one follicle had reached a diameter of 18 mm, and 34- 36 hours later, either standard IUI or FSP was performed. According to the collected data, including aged between<30 and 30-35 years old, primary or secondary infertility, and duration of infertility <5 or >5 years. On the day of hCG administration, the patients were randomly, according to a sealed envelope, divided into group I (n=45) to undergo standard IUI or group II (n=45) to undergo FSP using FAST system. The study was approved by Ethical Committee of the Institutional Review Board of Mazandaran University of Medical Sciences.

For all patients, semen was prepared by the standard swim-up technique. The final sperm suspension was diluted in 0/5 ml and 4 ml of Ham’s F-10 medium for IUI and FSP groups, respectively. In all cases, IUI and FSP were performed by a clinician and a technician. Intrauterine insemination was performed using the IUI catheter (Laboratoire C.C.D., Paris, France). The catheter was passed into the upper part of the uterine cavity, and 0.5 cc of sperm was slowly deposited. An air bubble was left behind the sperm suspension to provide complete delivery of the sperm suspension into the uterus. Patients rested for 30 minutes after insemination.

**Fig 1:**
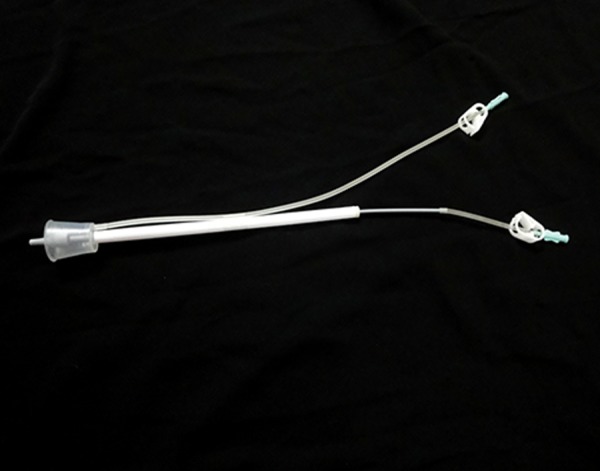
FAST System for fallopian tube sperm injection (FSP).

FSP was performed with FAST system’s catheter from the same company (Laboratoires C.C.D., Paris, France). This device is composed of a cervical cup made of crystal-clear plastic with two flexible tubings with a roller clamp on each, the injection tubing and the vacuum tubing (Fig 1) (16). Three different sizes of cervix adaptor were selected (diameters of 25, 27, and 30 mm) according to the size of the patient’s cervix. The syringe containing 4 cc processed semen was connected to the injection tubing. A sterile 10-mL syringe was connected to the vacuum tubing (16). Cervix was exposed by a bivalve speculum in lithotomy position, and the cervix was exposed and cleaned with physiological saline solution. According to the factory instruction, the cervical cup was grasped using a grasping forceps. Afterward, the adaptor was inserted into the vagina until the tip of the injection tubing entered into the cervix canal. Then, the edge of the cup was gently pressed into the cervix to make sure that it was in the right place. Furthermore, a vacuum was immediately created inside the adaptor by aspirating the syringe connected to the vacuum tubing. The sperm suspension was then slowly injected over 2 minutes (16). To push all the sperm that were in the dead space of the tubes into the uterus, the first syringe was disconnected and replaced with another sterile 5-mL syringe filled with 1.5 mL of incubation medium. FSP was completed by slowly injection of the medium. Then the tubing was attached to the inner thigh by means of a sticking plaster. After 2 hours, the device was easily removed with a gentle pull after opening the roller clamp of the vacuum tubing.

Patients received progesterone vaginal suppositories 400 mg per day (Cyclogest, Actavis, Iceland) for luteal-phase support. Patients were instructed to obtain a quantitative serum hCG 16- 18 days after insemination if no menses occurred. A transvaginal ultrasonogram was performed at 6-7 weeks after the last menstrual period to detect clinical pregnancy. A biochemical pregnancy was detected by a transient elevation of serum hCG.

### Statistical analysis

The Statistical Package for the Social Sciences (SPSS; SPSS Inc., Chicago, IL, USA) version 16.0 was used to assess the study data.We considered 20% pregnancy rate in IUI and 34% pregnancy rate in FSP with α=0.05 and β=0.2 using the following sample size formula:

n=Z1-α22P―1-P―+Z1-ßP11-P1+P21-P2P1-P22

Z=the standard normal variable unit, which at 95 percent is equal to 1.96.

P=proportion of the population trait. If not available, it can be considered 0/5.

The sample size assessed 90 patients. The twotailed t test and χ2 test were used for the statistical analysis. A p value of <0.05 was considered as significant difference.

## Results

Out of ninety patients with unexplained infertility enrolled in this study, 45 patients were randomly allocated to IUI group (group I) and 45 patients in FSP group (group II).

The median age values in the groups I and II were 28.2 ± 4.9 and 27.1 ± 4.6 years, respectively (p>0.05). Our findings shows that 74% in IUI group and 72% in FSP group had primary infertility (p>0.05). The mean duration values of infertility were 3.9 ± 3.1 years in group I and 3.8 ± 2 years in group II (p>0.05).The median of BMI values were 26.6 ± 2.7 kg/m^2^ in IUI group and 25.5 ± 2.3 kg/m^2^ in FSP group (p>0.05).The patients’ basic characteristics were not significantly different between the two study groups.

The characteristics of the stimulation cycles and outcome are presented in table 1. The numbers of follicles >16 mm during ovarian stimulation were 2.2 ± 1 in group I and 2.1 ± 0.9 in group II (p>0.05). The days of hCG administration in groups I and II were on 12.8 ± 3.4 and on 11.7 ± 2.6 of a cycle, respectively. The endometrial thickness values on the day of HCG administration were 8.2 ± 1 mm in group I and 8.8 ± 0.9 mm in group II. The mean numbers of motile spermatozoa inseminated were 49×10^6^ in group I and 51×10^6^ in group II. The cycle characteristics were not significantly different between the two study groups (p>0.05).

Clinical pregnancy rate values were 8 of 45 patients (17.8%) in the FSP group and 9 of 45 patients (20%) in the IUI group (p>0.05).

In both groups, insemination was easily performed in all patients, and no case of sperm reflux was observed. No complications such as cervical bleeding, vasovagal episodes, or uterine cramping were observed. No cases of ovarian hyperstimulation syndrome or cancellation of the cycle were observed.

**Table 1:** Characteristics of stimulation cycles and outcome in two studies groups


	Group I (IUI)	Group II (FSP)

**Number of patients**	45	45
**Number of cycles**	45	45
**Number of HMG ampules^¥^**	5.2 ± 2.2	5.4 ± 2.3
**Day of HCG administration^¥^**	12.8 ± 3.4	11.7 ± 2.6
**Number of follicles >16 mm^¥^**	2.2 ± 1	2.1 ± 0.9
**Thickness of endometrium (mm)^¥^**	8.2 ± 1	8.8 ± 0.9
**Inseminated progressive motile sperm count (×10^6^)**	49	51
**Clinical pregnancies (%)**	9/45 (20%)	8/45 (17.8%)
**Abortion**	0	0
**Ectopic pregnancy**	0	0
**Multiple pregnancies**	1	0


¥; Values are presented as mean± SD, HMG; Human menopausal gonadotropin, IUI; Intrauterine insemination and FSP; Fallopian tube sperm perfusion. All p values are >0.05.

## Discussion

In this prospective randomized study, we compared the relative efficacy between FSP using FAST system and IUI in unexplained infertility population. We demonstrated no statistically significant difference between both treatment group in the pregnancy rate (17.8% in FSP vs. 20% in IUI groups) (p>0.05).The pregnancy rate with FSP is less than that reported by Kahn et al. (10), they reported 26.9% pregnancy rate in the FSP group versus9.8% in the IUI group (p<0.05).

In Fanchin and colleagues’study (1), FSP was performed using an auto-blocking device (FAST system) similar to our study. Fanchin reported 40% pregnancy rate per cycle in FSP group versus 20% in the IUI group (p<0.05), however, they failed to determine the cause of infertility in their patients (17). Theoretically, the direct passage of the sperm preparation through the fallopian tubes would increase the density of capacitated spermatozoids near the oocyte and the intra-peritoneal cavity and by consequence increase the pregnancy success rate (11).The pressure injection of inseminate in FSP can remove partial obstruction of fallopian tubes, created by thick mucus or tubal polyps (1). Some authors reported pregnancy rates of 20-40 % in FSP technique (1, 8, 10, 15). One metaanalysis study by Trout and Kemman (17) demonstrated a significant difference of superiority for FSP concerning the unexplained infertility, 22% of pregnancy rate in FSP versus 13% in IUI. They included all the previous studies from 1992 to 1998, but exempted Fanchin et al. (1) who didn’t detail their indications and results. Trout and Kemman’s meta-analysis showed a significant improvement in pregnancy rates with FSP only in patients with unexplained infertility who underwent controlled ovarian stimulation with gonadotropin and insemination protocols (17). We used clomiphene and gonadotropin combination for induction of ovulation to reduce the cost of treatment. Selecting the different induction ovulation protocol may explain the differences between their and our findings. In El-Khayat and colleagues’ study, the pregnancy rate was significantly higher in FSP group than in IUI group (26.7 vs. 11.7%, respectively, p<0.04). They achieved FSP via Foley catheter with 4 mL of inseminate in patients with mild or moderate male factor infertility (12).

In contrast, other authors have reported the pregnancy rate of 9 or 14.5% in FSP technique (20, 21). The results of Panayotidis’s study didn’t show a statistically significant superiority of the FSP over the IUI method for all the indications of insemination (11). Our results are similar to Nuojua- Huttunen and colleagues’ study. They performed a prospective randomized study using a Foley catheter for FSP. They reported no advantage of FSP in comparison with the conventional IUI technique in women with unexplained infertility, minimal to mild endometriosis, mild male factor, and ovarian dysfunction. However, the Foley catheter is cheaper, but sometimes, there is difficult to introduce this tool into the cervical canal. It might have an adverse effect on the endometrium caused by pressure of the balloon and the substances that may dissolved from the Foley catheter (19). Only truly randomized controlled studies comparing FSP with IUI were included in this review. Eight studies involving 595 couples were included in the meta-analysis. Only one study reported the live birth rate and there was no evidence of a difference between FSP and IUI (OR 1.2, 95% CI: 0.39 to 3.5). There was no evidence of a difference between FSP and IUI for clinical pregnancy per couple (OR 1.2, 95% CI: 0.79-1.7). A subgroup analysis including couples with unexplained subfertility did not report any difference between FSP and IUI (OR 1.6, 95% CI: 0.89-2.8) (22).

Since 1992, the following different protocols for ovarian stimulation are applied: clomiphene, alone or combined with FSH, and HMG. Different protocol for induction ovulation is one of the factors that could explain differences in results. Another factor to explain the differences in result is the type of catheter used to place sperm in the fallopian tube. As mentioned in studies, the use of the FAST system for FSP can be a little more expensive than the IUI catheter, and sometimes, the placement of the seal cup on the cervix is not perfect and needs more experience and skill (16). In our clinic, we perform routinely FSP with Foley catheter and FAST system, so skill of clinician can’t be considered as a factor for difference.

From this study we conclude that FSP using FAST system offers no advantage over the standard IUI in order to increase the pregnancy rate in unexplained infertility. The FSP technique needs more media volume for insemination, so the procedure could be more expensive. Maher Shams evaluated the efficacy of double FSP versus single FSP by Foley catheter in non-tubal infertility. They showed higher pregnancy rate in double FSP groups (23). Doing double FSP in unexplained infertility could be a topic for our future study.

## Conclusion

Future well-designed study in larger population is needed to confirm benefits of FSP. We suggest double FSP or tubo-peritoneal perfusion with 10 ml of inseminated before using other more expensive and invasive assisted reproductive technique in unexplained infertility patients.
